# Supplementary material for: Model-based assessment of public health impact and cost-effectiveness of dengue vaccination following screening for prior exposure
Source: PLoS Negl Trop Dis. 2019 Jul 1;13(7):e0007482. doi: 10.1371/journal.pntd.0007482 (PMC6625736; doi:10.1371/journal.pntd.0007482)
Supplement: S1 Appendix — (PDF) [file pntd.0007482.s001.pdf]

Appendix S1. Process for achieving a desired value of PE_9 in model simulations.

To afford the model the flexibility to achieve a range of transmission intensities, as defined by PE_9, we developed a statistical emulator of PE_9 as a function of three unknown model parameters (rate at which DENV is seeded into the population, mosquito infectiousness, adult female mosquito emergence rate). To do so, we generated 10^3 combinations of these three parameters using the sobol function in the pomp library [25] in R [26]. This function generates points that maximize distance between them within a prescribed range of values for each parameter (DENV seeding rate: $8x10^{-6}$-$2x10^{-4}$; mosquito infectiousness: 0-1; adult female mosquito emergence rate: 0-3). After simulating 40 years of transmission with a given set of those three parameters, we retrieved PE_9 from all such simulations and fitted a generalized additive model of PE_9 with independent smooth terms for each of the three parameters ($R^2 = 0.98$). In subsequent simulations focused on vaccination impact, we obtained a set of the three unknown model parameters consistent with a target value of PE_9 by repeatedly drawing sets of the three parameters until we obtained one that was associated with a value of PE_9 within one percent of the target PE_9 value.
