# Supplementary material for: Model-based assessment of public health impact and cost-effectiveness of dengue vaccination following screening for prior exposure
Source: PLoS Negl Trop Dis. 2019 Jul 1;13(7):e0007482. doi: 10.1371/journal.pntd.0007482 (PMC6625736; doi:10.1371/journal.pntd.0007482)
Supplement: S2 Appendix — (PDF) [file pntd.0007482.s002.pdf]

Appendix S2. Statistical emulator of vaccination impact projections.

Despite efforts to minimize stochastic differences between paired simulations under a given parameter set, the proportions of cases averted resulting from our simulations were relatively noisy. This is due to the dynamics of the model, which are characterized by large epidemics in some years separated by low levels of incidence during inter-epidemic periods [20]. Thus, even small differences in the sequence of random number draws (due to differences in infection outcomes associated with protective effects of vaccination) can lead otherwise similar pairs of simulations to diverge in their behavior over time. Even so, there were clear patterns in the central tendency of the proportion of cases averted as a function of the parameters varied across the 10^3 parameter sets that we examined. To extract pattern from noise, we developed a statistical emulator of the proportion of cases averted as a function of four parameters described in the previous paragraph using the randomForest function from the randomForest library [49] in R. Values of the proportion of cases averted from this emulator were likewise used in calculations of cost-effectiveness.
