# Supplementary material for: Model-based assessment of public health impact and cost-effectiveness of dengue vaccination following screening for prior exposure
Source: PLoS Negl Trop Dis. 2019 Jul 1;13(7):e0007482. doi: 10.1371/journal.pntd.0007482 (PMC6625736; doi:10.1371/journal.pntd.0007482)
Supplement: S4 Appendix — (PDF) [file pntd.0007482.s004.pdf]

Appendix S4. Estimate of the price of Dengvaxia in the Philippines.

In 2016, the Philippines government paid a total of P3.5 billion to vaccinate a total of 1,077,623 9-year-old public-school students [34]. We assumed that this cost allowed for three doses of vaccine plus the cost of administrating it. Hence, the unit price of a fully vaccinated person was around P3,247. This corresponded to 69.3 USD in 2016, which we rounded to 69 USD. This cost can be recalculated and updated in our analyses using the web application available online.
