# Supplementary figures and images for: Model-based assessment of public health impact and cost-effectiveness of dengue vaccination following screening for prior exposure
Source: PLoS Negl Trop Dis. 2019 Jul 1;13(7):e0007482. doi: 10.1371/journal.pntd.0007482 (PMC6625736; doi:10.1371/journal.pntd.0007482)

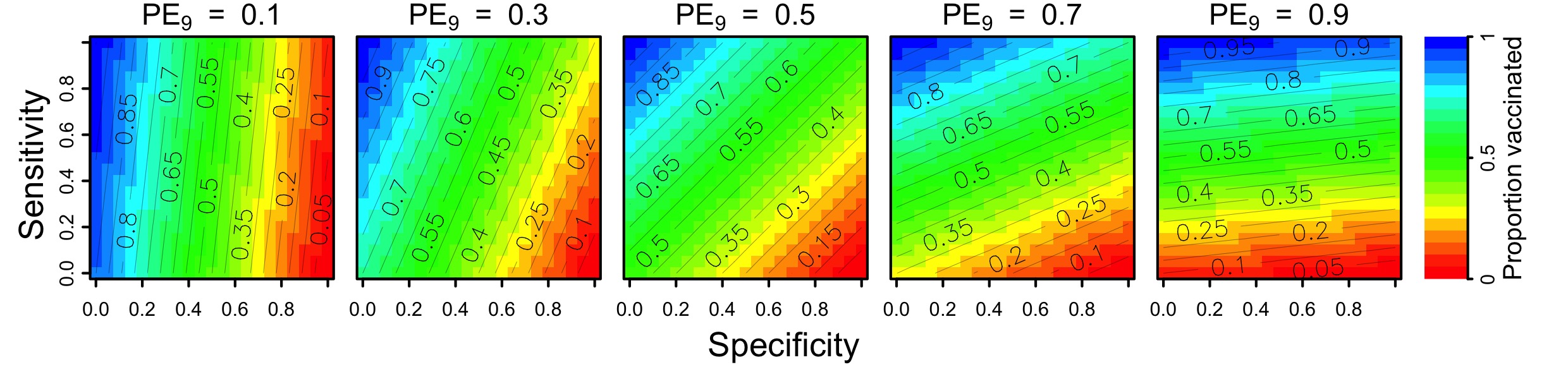

Supplement: S1 Fig — This relationship depends on the sensitivity (y-axis) and specificity (x-axis) of serological screening. (JPEG) [file pntd.0007482.s005.jpeg]

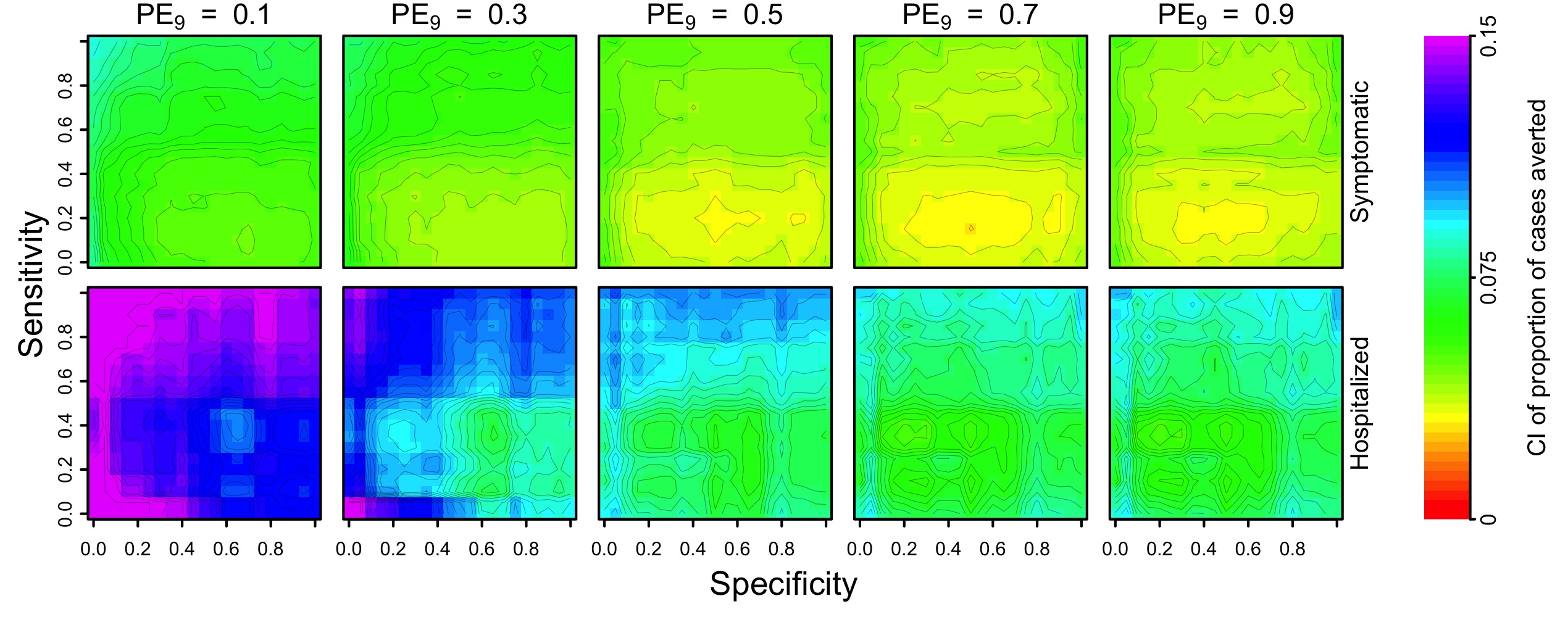

Supplement: S2 Fig — Width of the confidence interval of the cumulative proportion of cases averted over a 30-year period (top row: symptomatic, bottom row: hospitalized) as a function of the sensitivity (y-axis) and specificity (x-axis) of serological screening. Each column shows these results in a given transmission setting, defined by PE9. (JPEG) [file pntd.0007482.s006.jpeg]

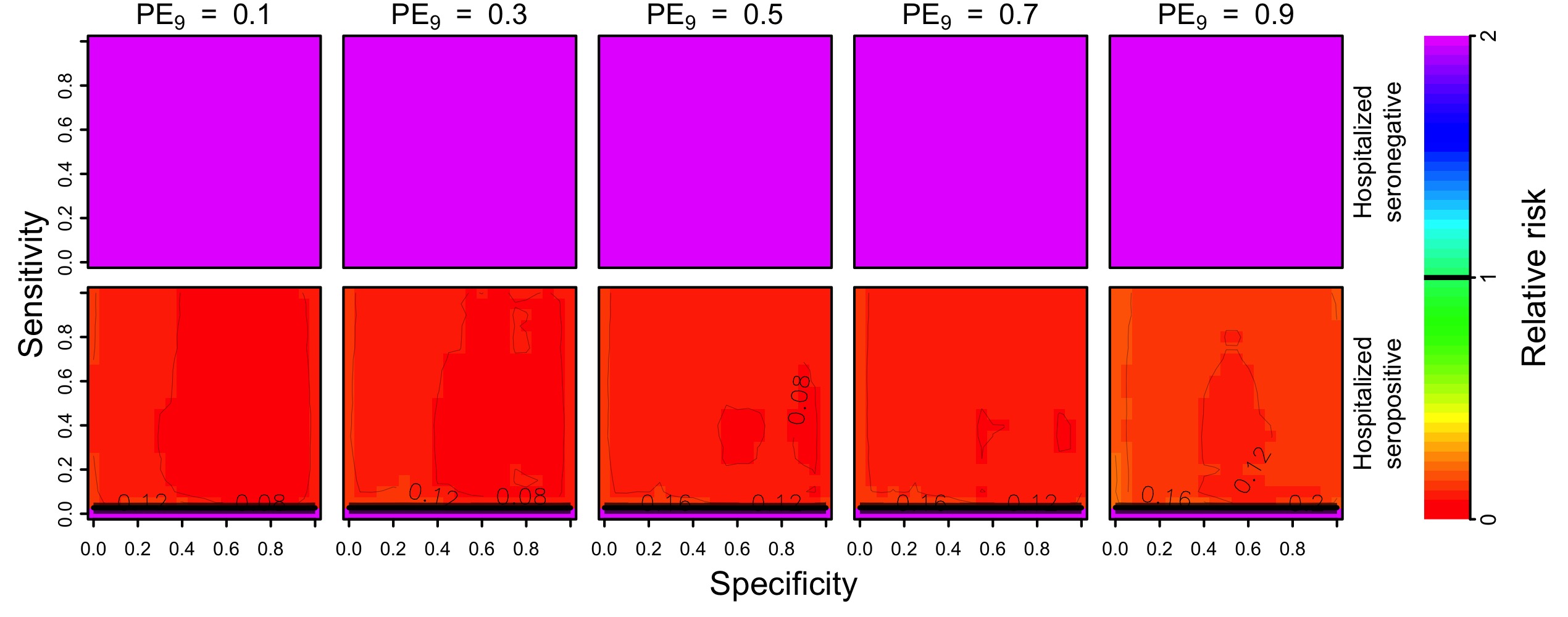

Supplement: S3 Fig — Per capita relative risk (colors) of hospitalization for individuals seronegative (top) and seropositive (bottom) over a 10-year horizon in the first cohort of individuals eligible for vaccination after a positive result from serological screening, as a function of the sensitivity (y-axis) and specificity (x-axis) of serological screening. Each column shows these results in a given transmission setting, defined by the proportion of nine-year-olds with previous DENV exposure, PE9. (JPEG) [file pntd.0007482.s007.jpeg]

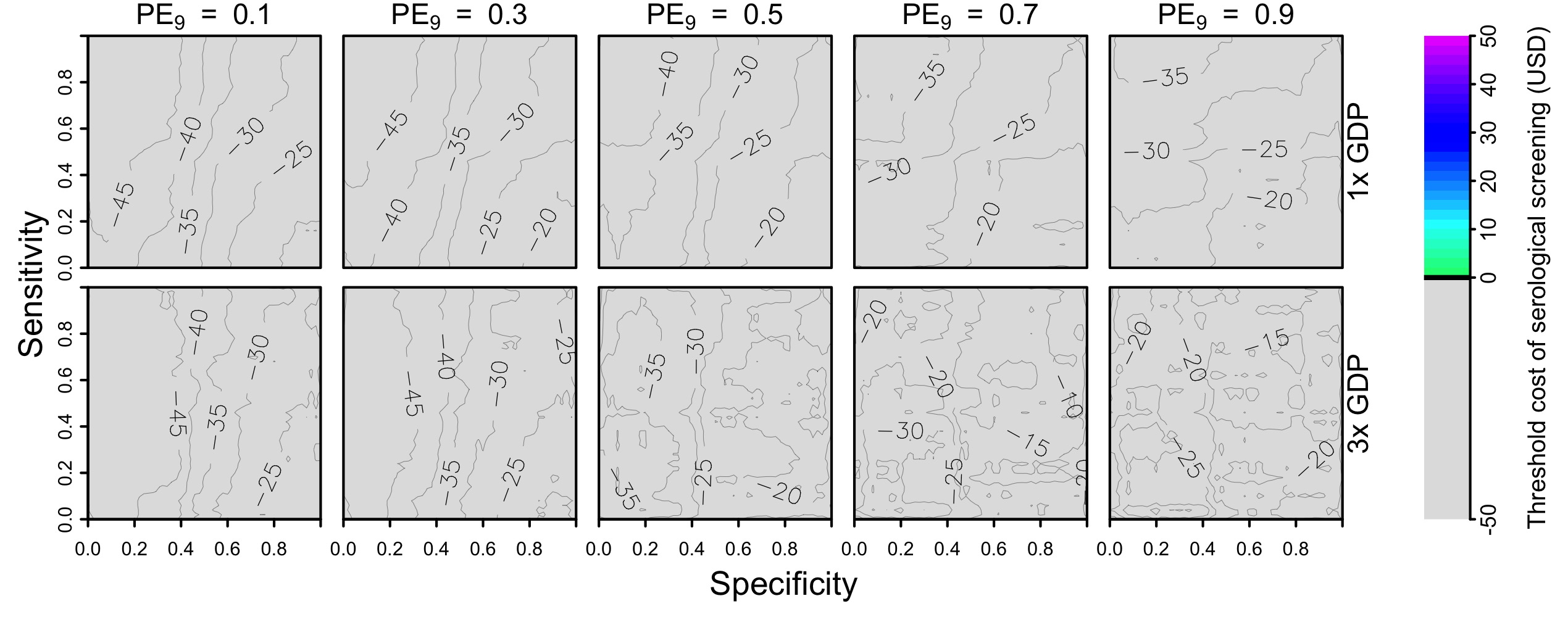

Supplement: S4 Fig — (JPEG) [file pntd.0007482.s008.jpeg]

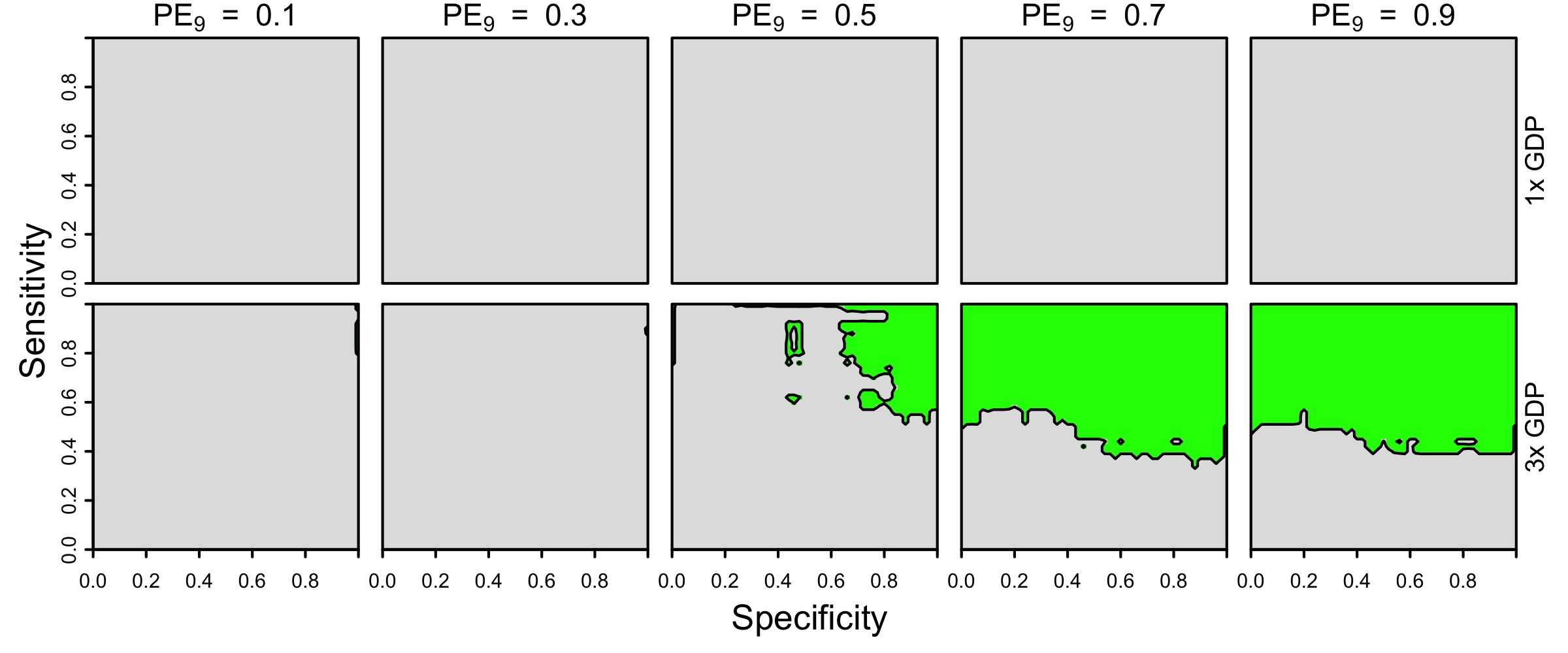

Supplement: S5 Fig — Threshold costs are indicated by color as a function of sensitivity (y-axis), specificity (x-axis), and PE9 value (columns). The value of costDALY is equal to per capita GDP (2,951 USD) in the top row and three times per capita GDP in the bottom row. (JPEG) [file pntd.0007482.s009.jpeg]

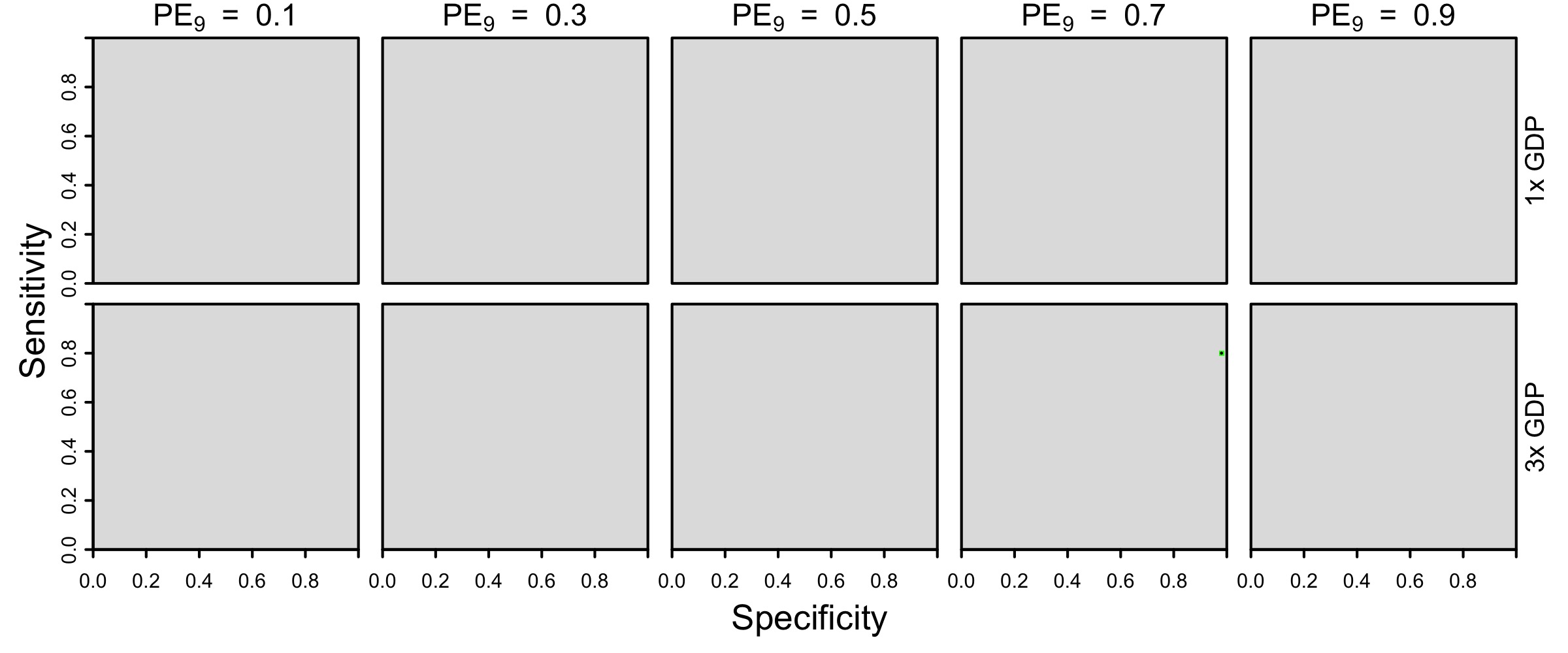

Supplement: S6 Fig — Cost-effectiveness according to Eq 5 is shown in green as a function of sensitivity (y-axis), specificity (x-axis), and PE9 value (columns). The value of costDALY is equal to per capita GDP (8,650 USD) in the top row and three times per capita GDP in the bottom row. (JPEG) [file pntd.0007482.s010.jpeg]

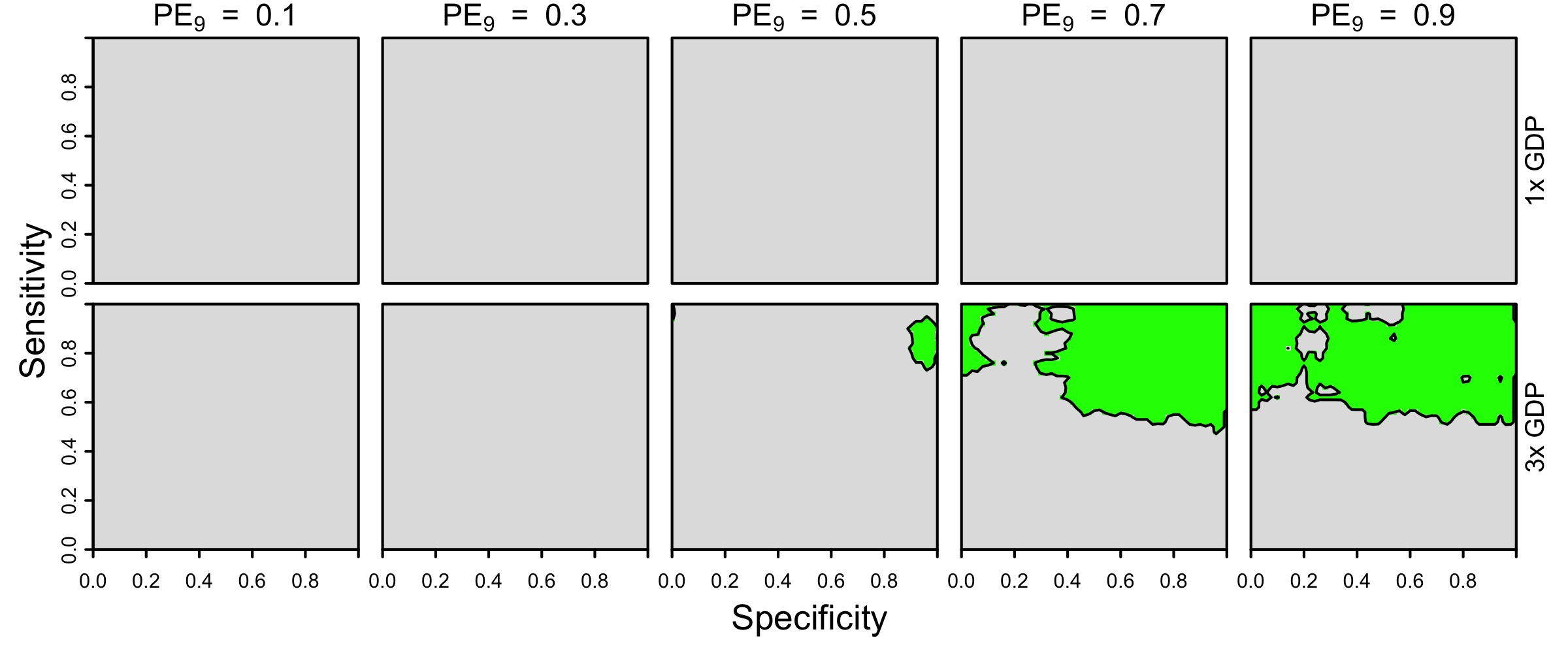

Supplement: S7 Fig — Cost-effectiveness according to Eq 5 is shown in green as a function of sensitivity (y-axis), specificity (x-axis), and PE9 value (columns). The value of costDALY is equal to per capita GDP (2,951 USD) in the top row and three times per capita GDP in the bottom row. (JPEG) [file pntd.0007482.s011.jpeg]

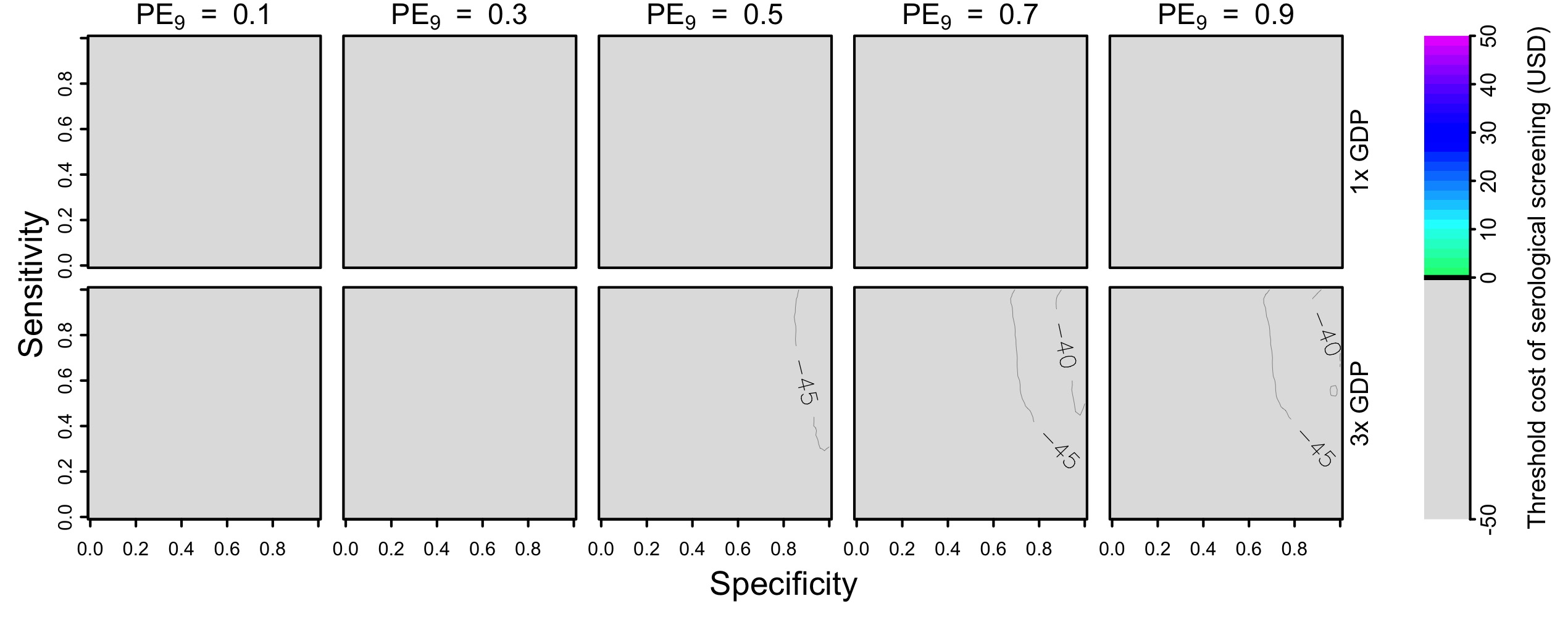

Supplement: S8 Fig — Cost-effectiveness according to Eq 5 is shown in green as a function of sensitivity (y-axis), specificity (x-axis), and PE9 value (columns). The value of costDALY is equal to per capita GDP (2,951 USD) in the top row and three times per capita GDP in the bottom row. (JPEG) [file pntd.0007482.s012.jpeg]

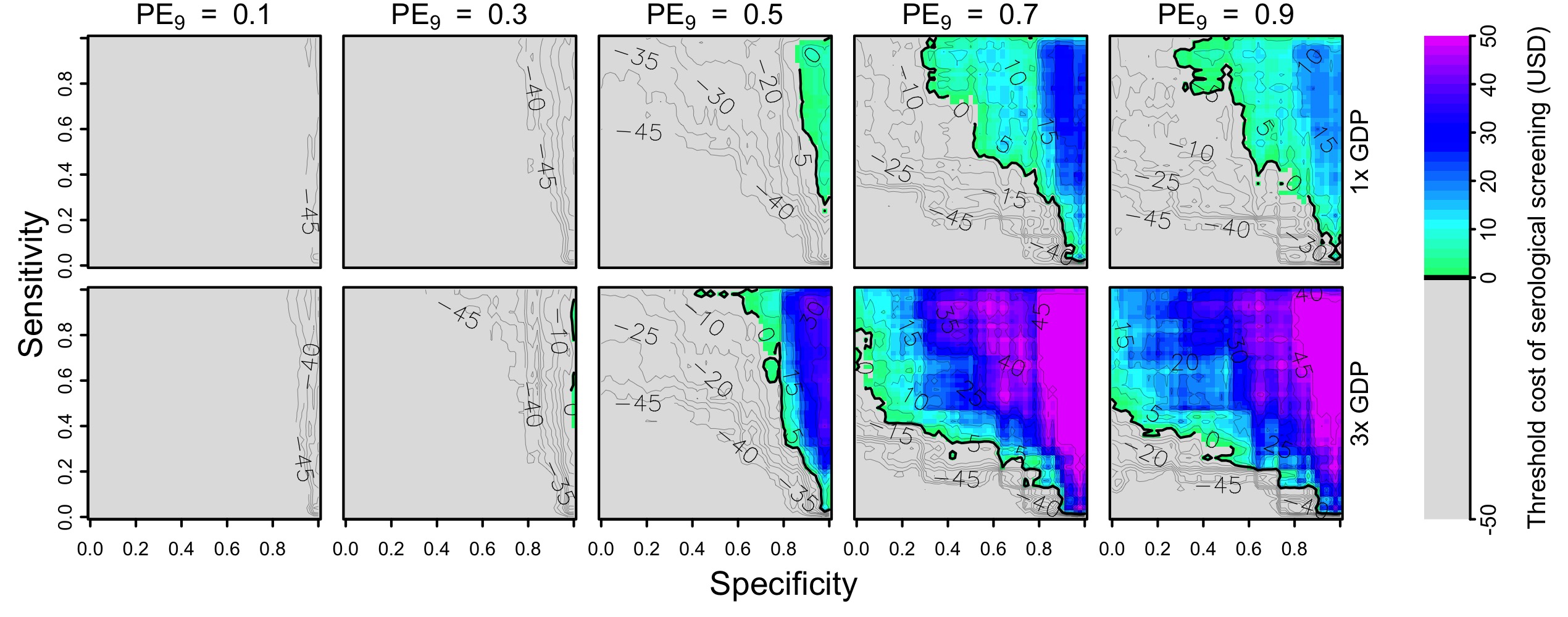

Supplement: S9 Fig — Threshold costs are indicated by color as a function of sensitivity (y-axis), specificity (x-axis), and PE9 value (columns). The value of costDALY is equal to per capita GDP (2,951 USD) in the top row and three times per capita GDP in the bottom row. (JPEG) [file pntd.0007482.s013.jpeg]

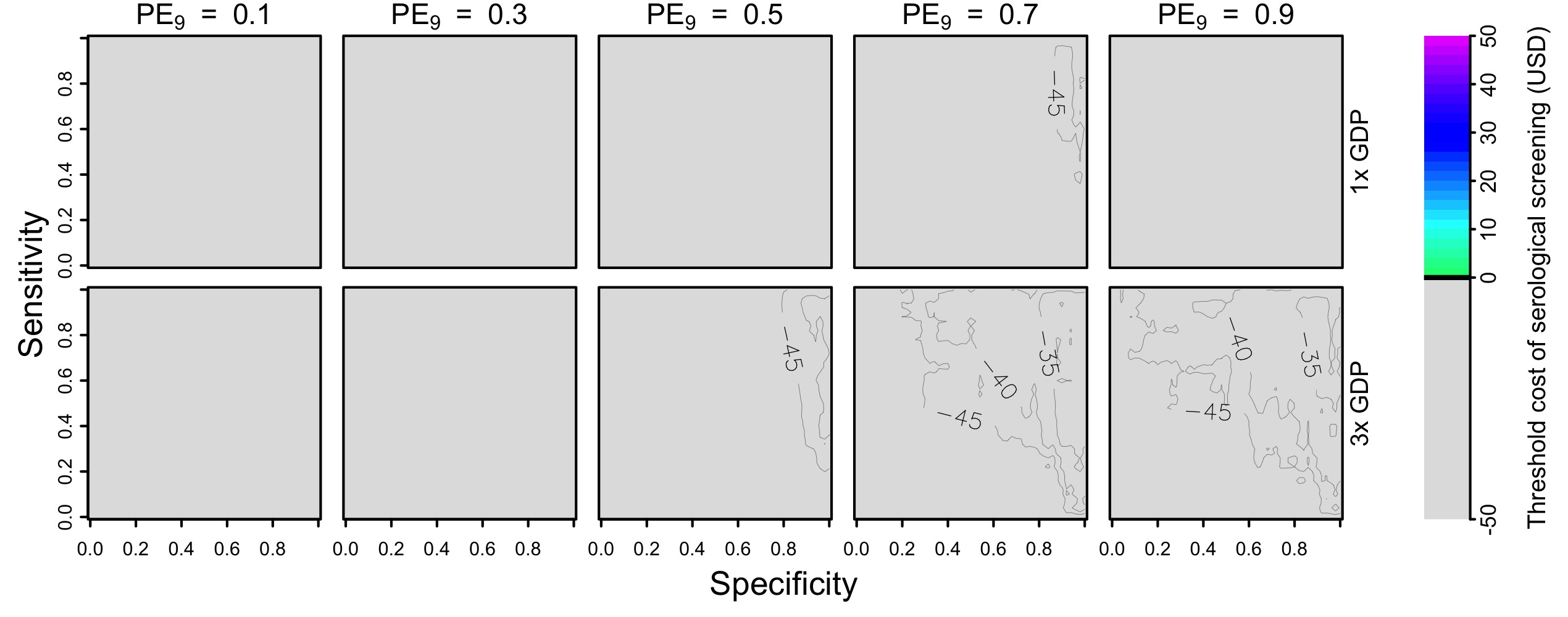

Supplement: S10 Fig — Cost-effectiveness according to Eq 5 is shown in green as a function of sensitivity (y-axis), specificity (x-axis), and PE9 value (columns). The value of costDALY is equal to per capita GDP (8,650 USD) in the top row and three times per capita GDP in the bottom row. (JPEG) [file pntd.0007482.s014.jpeg]

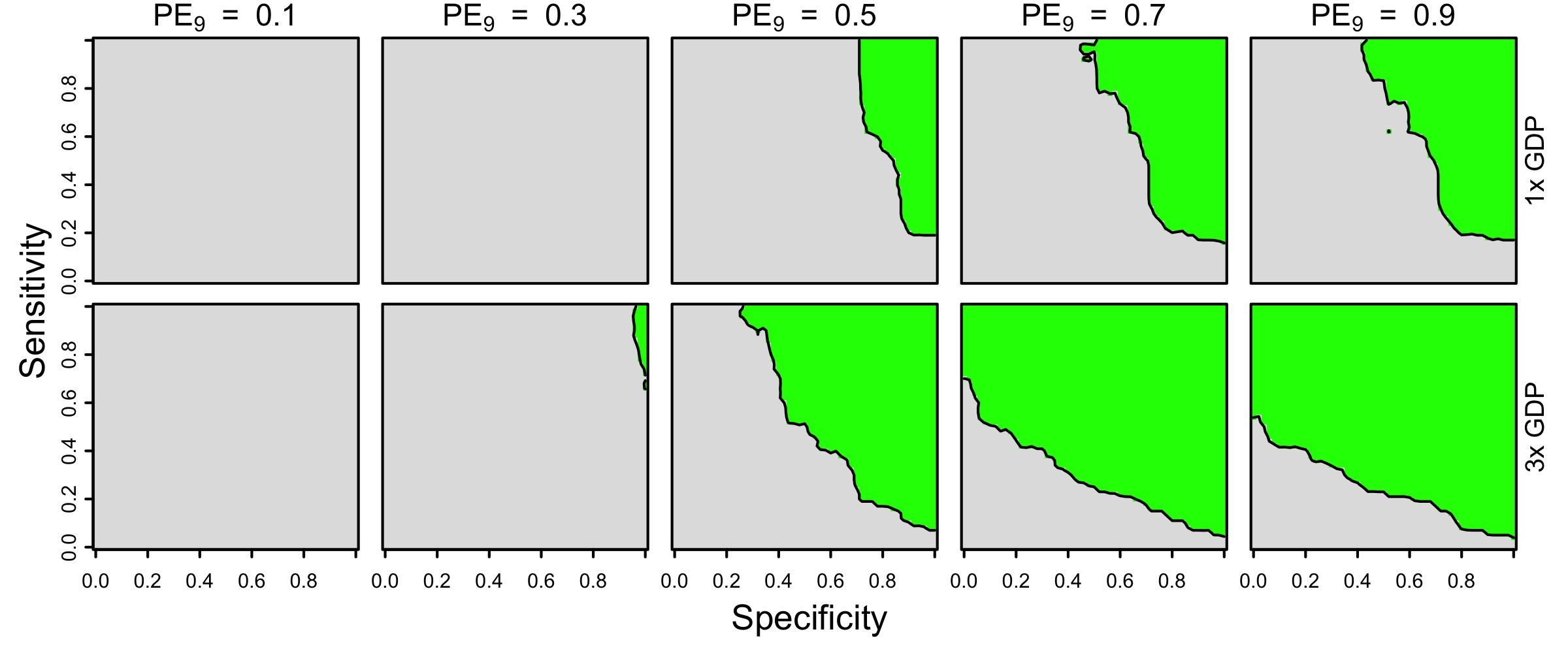

Supplement: S11 Fig — Cost-effectiveness according to Eq 5 is shown in green as a function of sensitivity (y-axis), specificity (x-axis), and PE9 value (columns). The value of costDALY is equal to per capita GDP (2,951 USD) in the top row and three times per capita GDP in the bottom row. (JPEG) [file pntd.0007482.s015.jpeg]

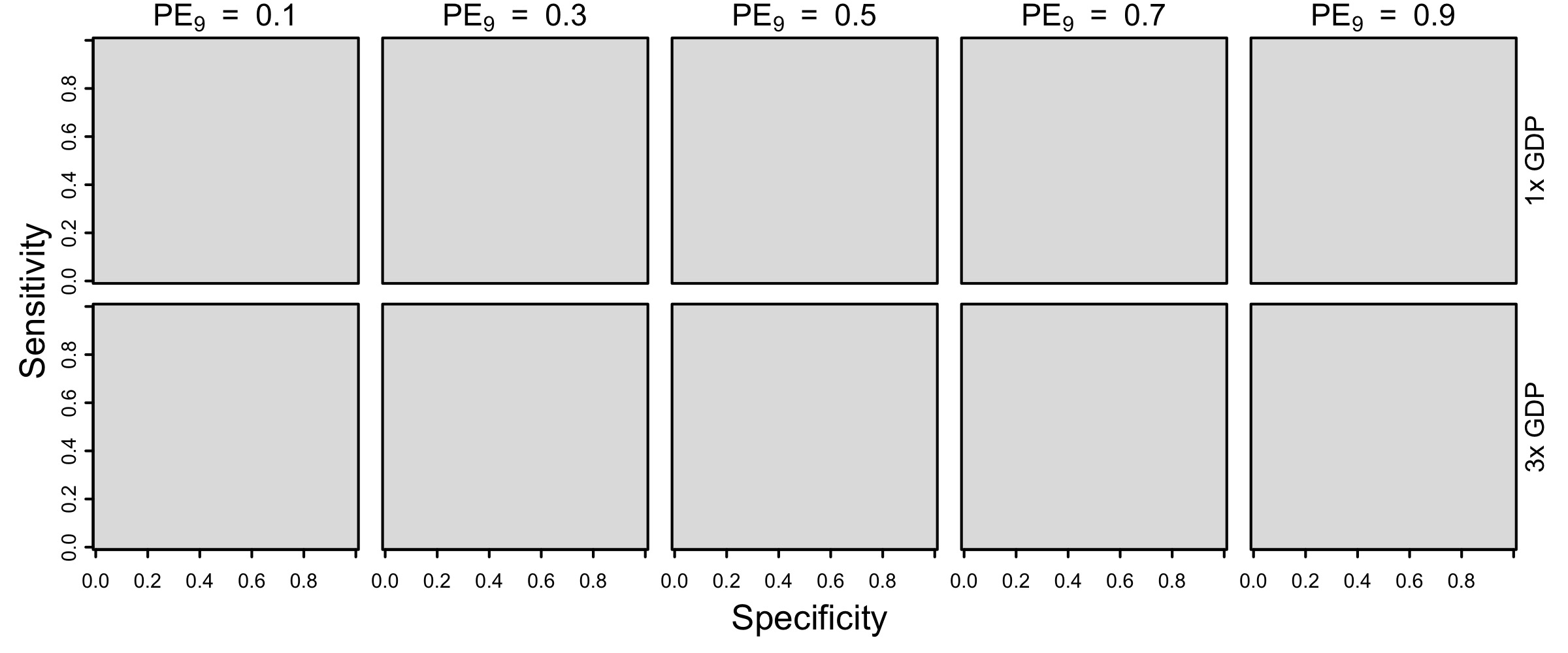

Supplement: S12 Fig — Cost-effectiveness according to Eq 5 is shown in green as a function of sensitivity (y-axis), specificity (x-axis), and PE9 value (columns). The value of costDALY is equal to per capita GDP (8,650 USD) in the top row and three times per capita GDP in the bottom row. (JPEG) [file pntd.0007482.s016.jpeg]

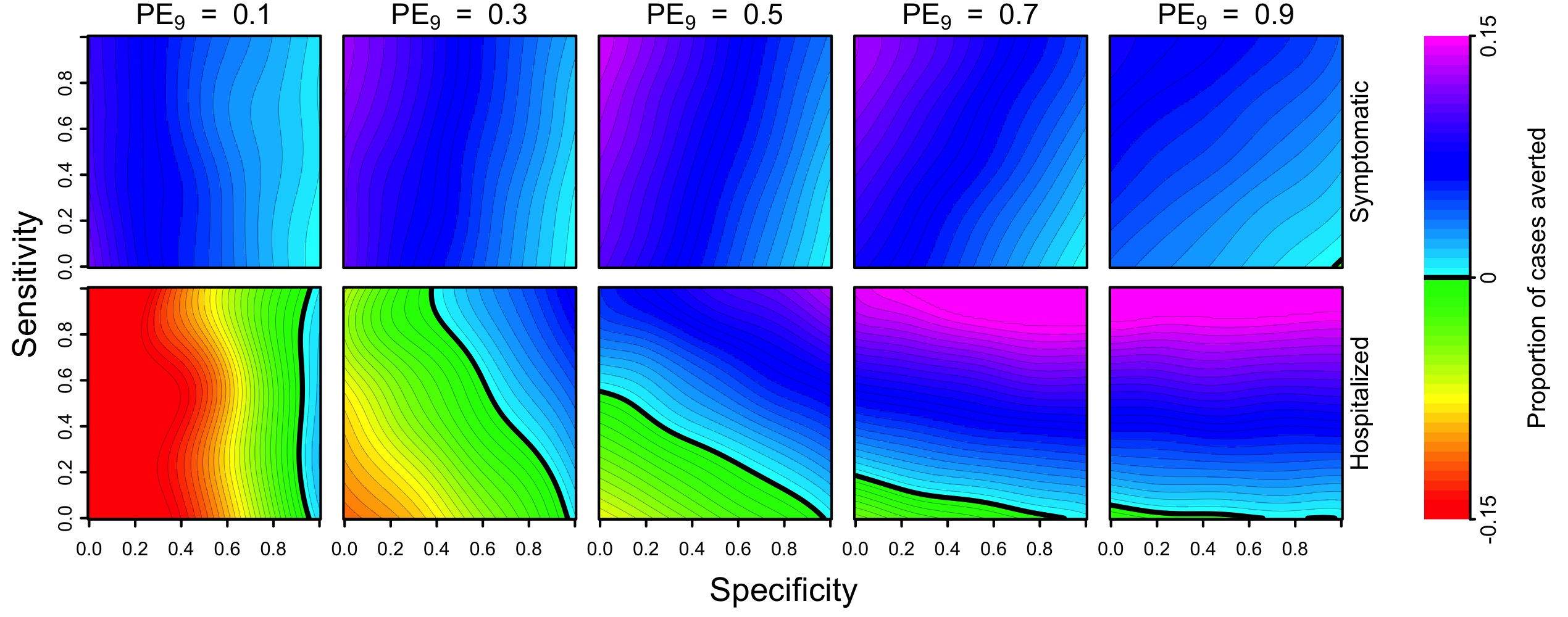

Supplement: S15 Fig — Cumulative proportion of cases averted (colors) over a 30-year period (top: symptomatic, bottom: hospitalized) as a function of the sensitivity (y-axis) and specificity (x-axis) of serological screening. Each column shows results for a given transmission setting, defined by the proportion of nine-year-olds with previous DENV exposure, PE9. (JPEG) [file pntd.0007482.s019.jpeg]

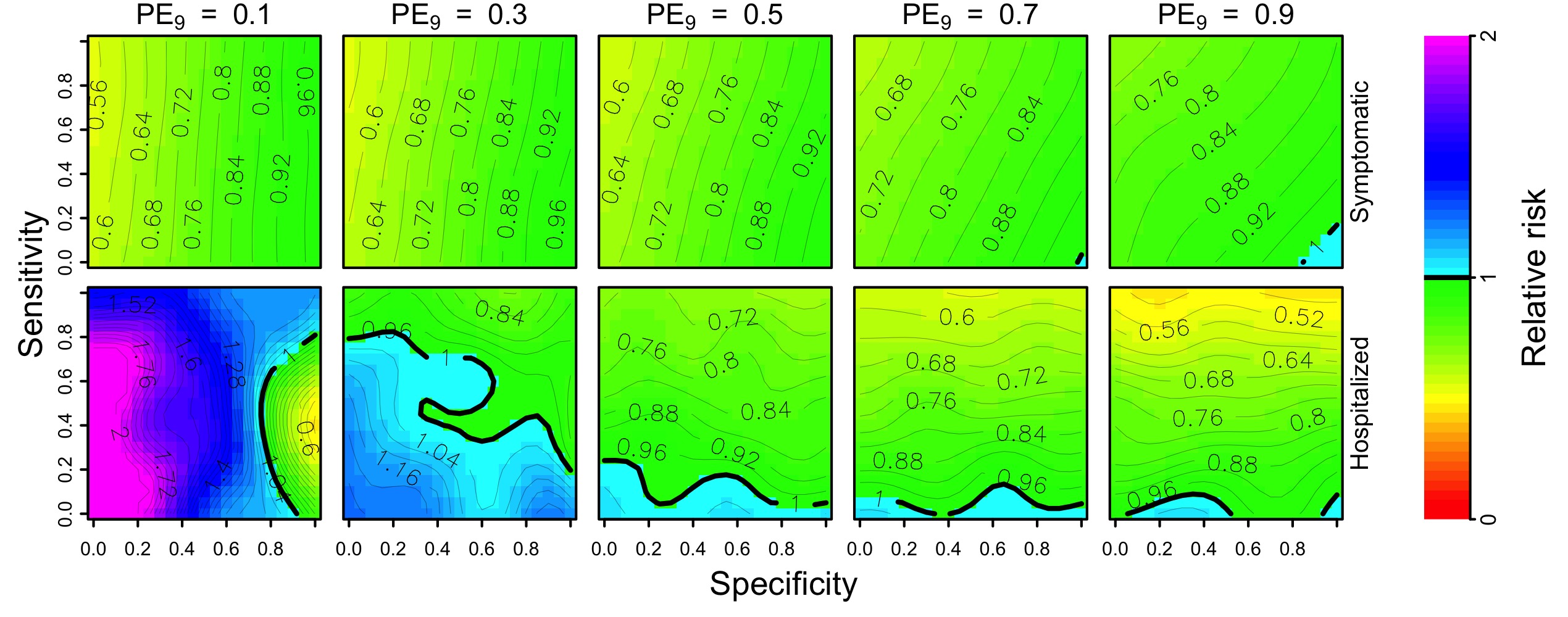

Supplement: S16 Fig — Per capita relative risk (colors) of symptomatic (top) and hospitalized (bottom) disease over a 30-year horizon in the first cohort eligible for vaccination after serological screening with a positive result, as a function of the sensitivity (y-axis) and specificity (x-axis) of serological screening. Each column shows these results in a given transmission setting, defined by the proportion of nine-year-olds with previous DENV exposure, PE9. (JPEG) [file pntd.0007482.s020.jpeg]

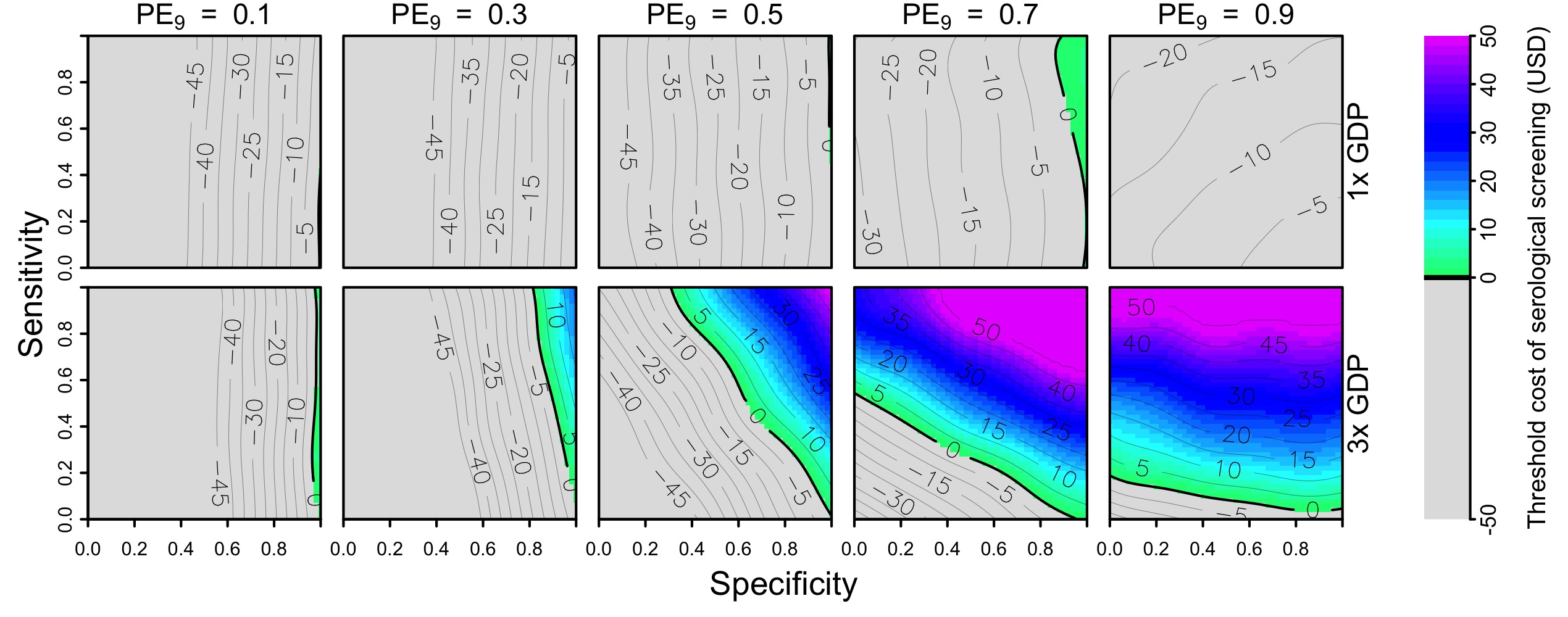

Supplement: S17 Fig — Threshold costs are indicated by color as a function of sensitivity (y-axis), specificity (x-axis), and PE9 value (columns). The value of costDALY is equal to per capita GDP (8,650 USD) in the top row and three times per capita GDP in the bottom row. (JPEG) [file pntd.0007482.s021.jpeg]

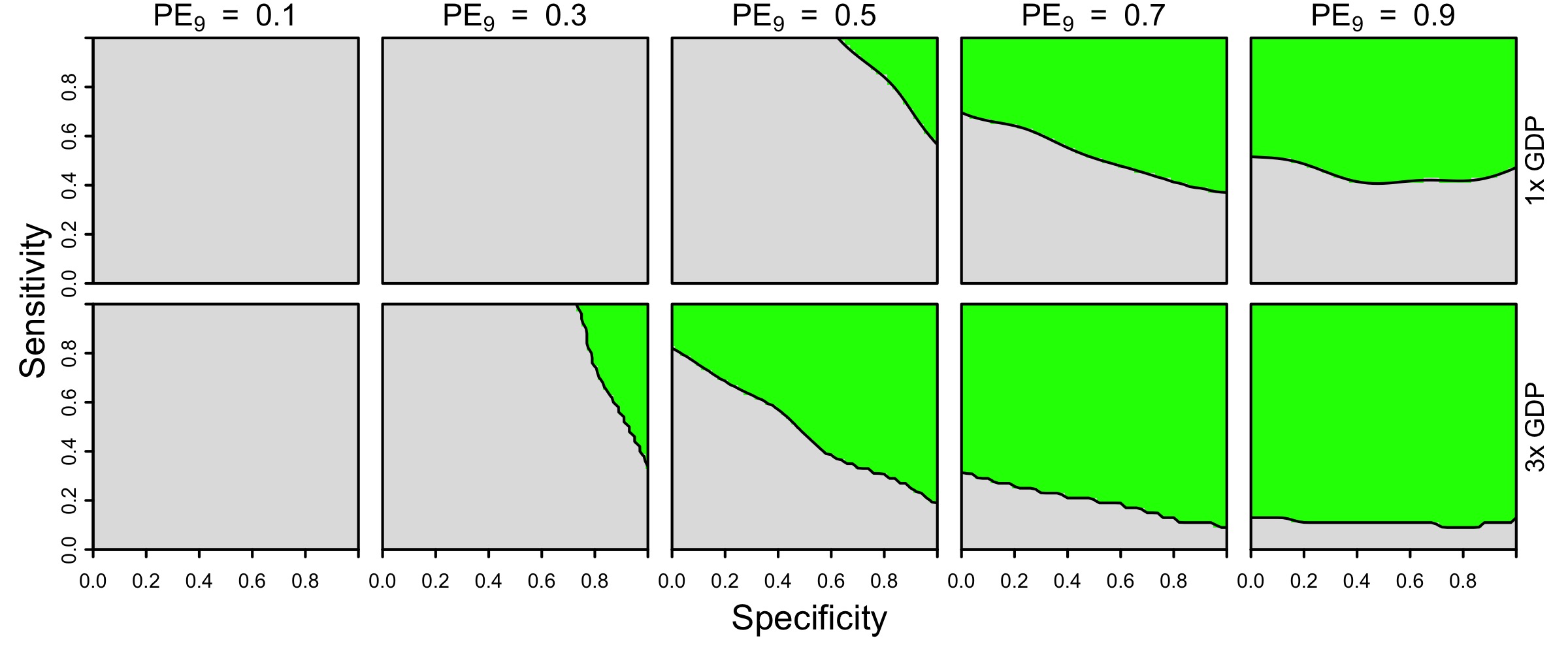

Supplement: S18 Fig — Cost-effectiveness according to Eq 5 is shown in green as a function of sensitivity (y-axis), specificity (x-axis), and PE9 value (columns). The value of costDALY is equal to per capita GDP (8,650 USD) in the top row and three times per capita GDP in the bottom row. (JPEG) [file pntd.0007482.s022.jpeg]

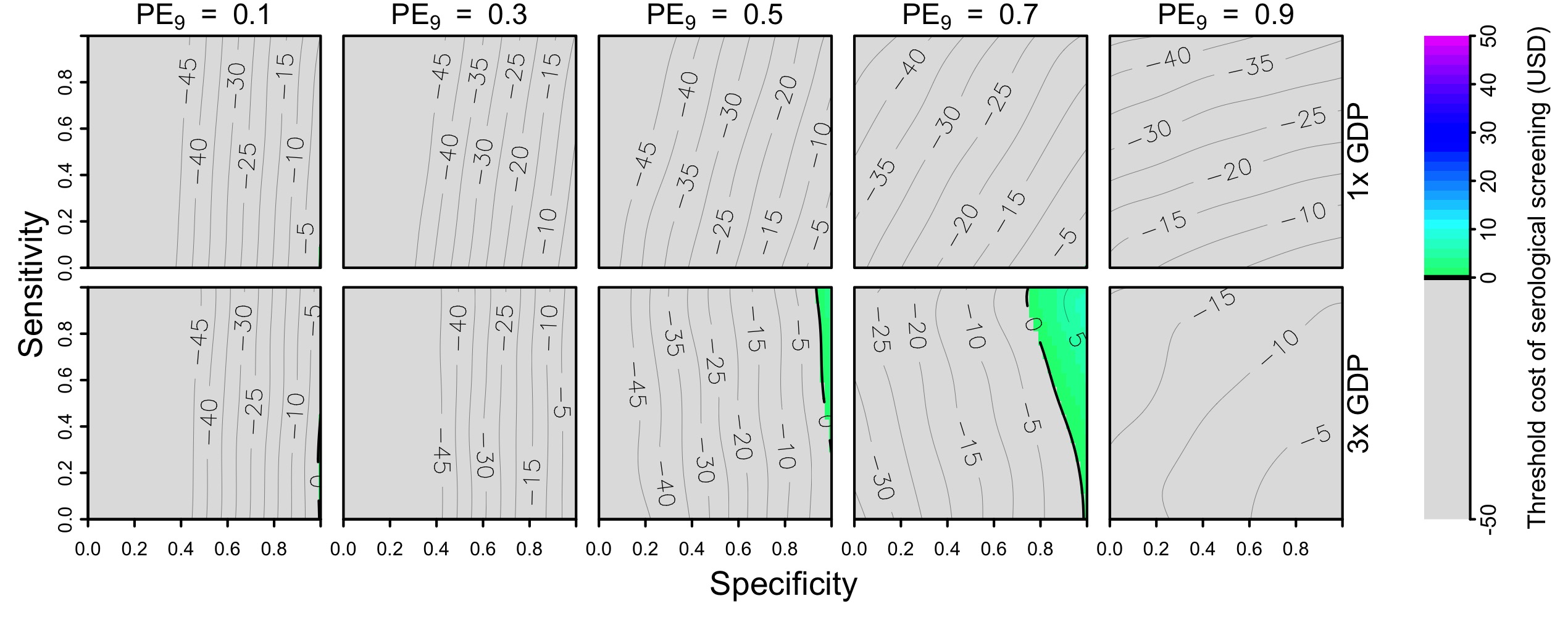

Supplement: S19 Fig — Threshold costs are indicated by color as a function of sensitivity (y-axis), specificity (x-axis), and PE9 value (columns). The value of costDALY is equal to per capita GDP (8,650 USD) in the top row and three times per capita GDP in the bottom row. (JPEG) [file pntd.0007482.s023.jpeg]

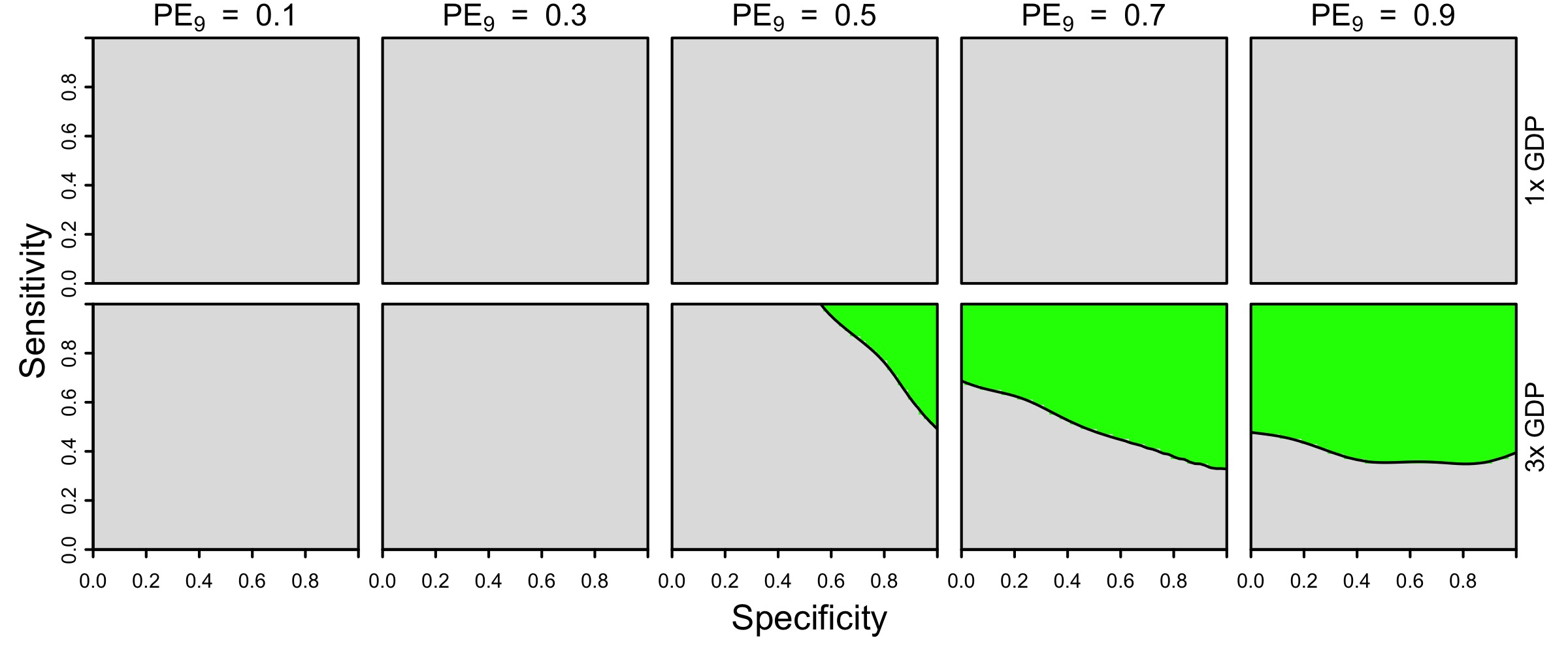

Supplement: S20 Fig — Cost-effectiveness according to Eq 5 is shown in green as a function of sensitivity (y-axis), specificity (x-axis), and PE9 value (columns). The value of costDALY is equal to per capita GDP (8,650 USD) in the top row and three times per capita GDP in the bottom row. (JPEG) [file pntd.0007482.s024.jpeg]

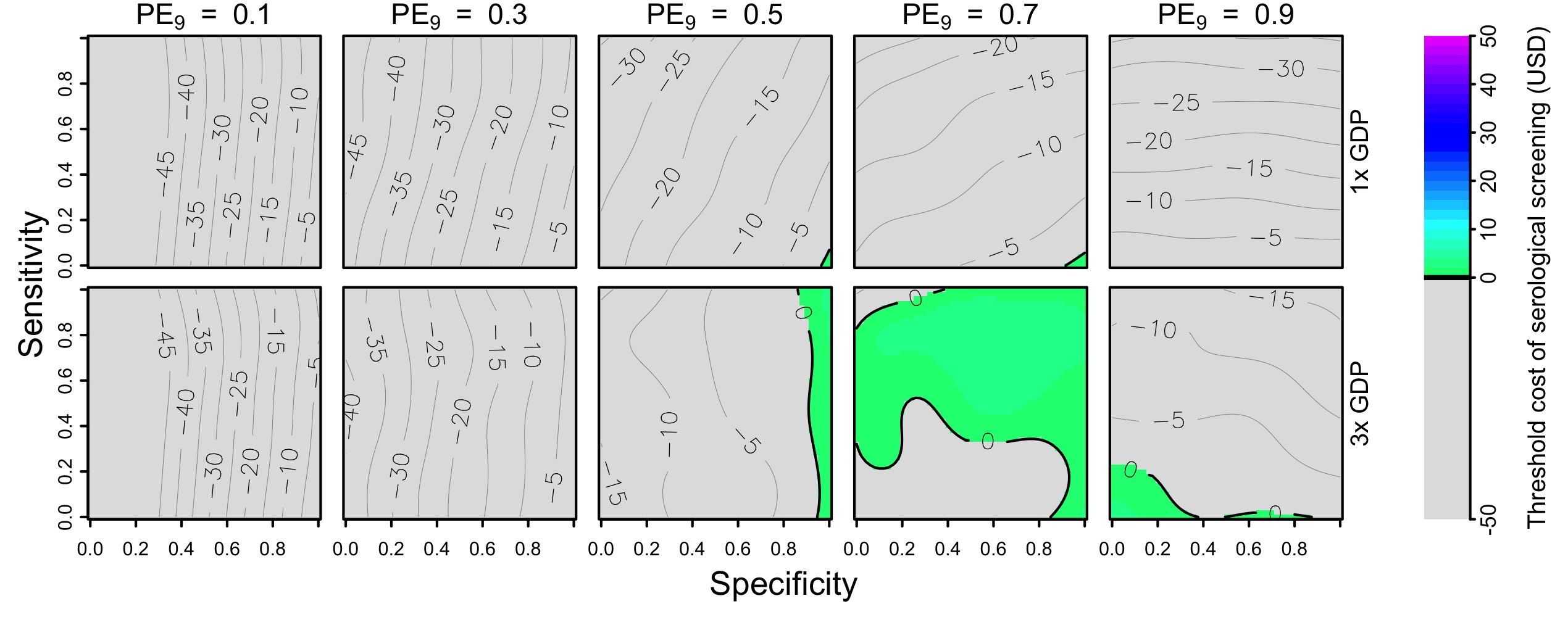

Supplement: S21 Fig — Threshold costs are indicated by color as a function of sensitivity (y-axis), specificity (x-axis), and PE9 value (columns). The value of costDALY is equal to per capita GDP (8,650 USD) in the top row and three times per capita GDP in the bottom row. (JPEG) [file pntd.0007482.s025.jpeg]

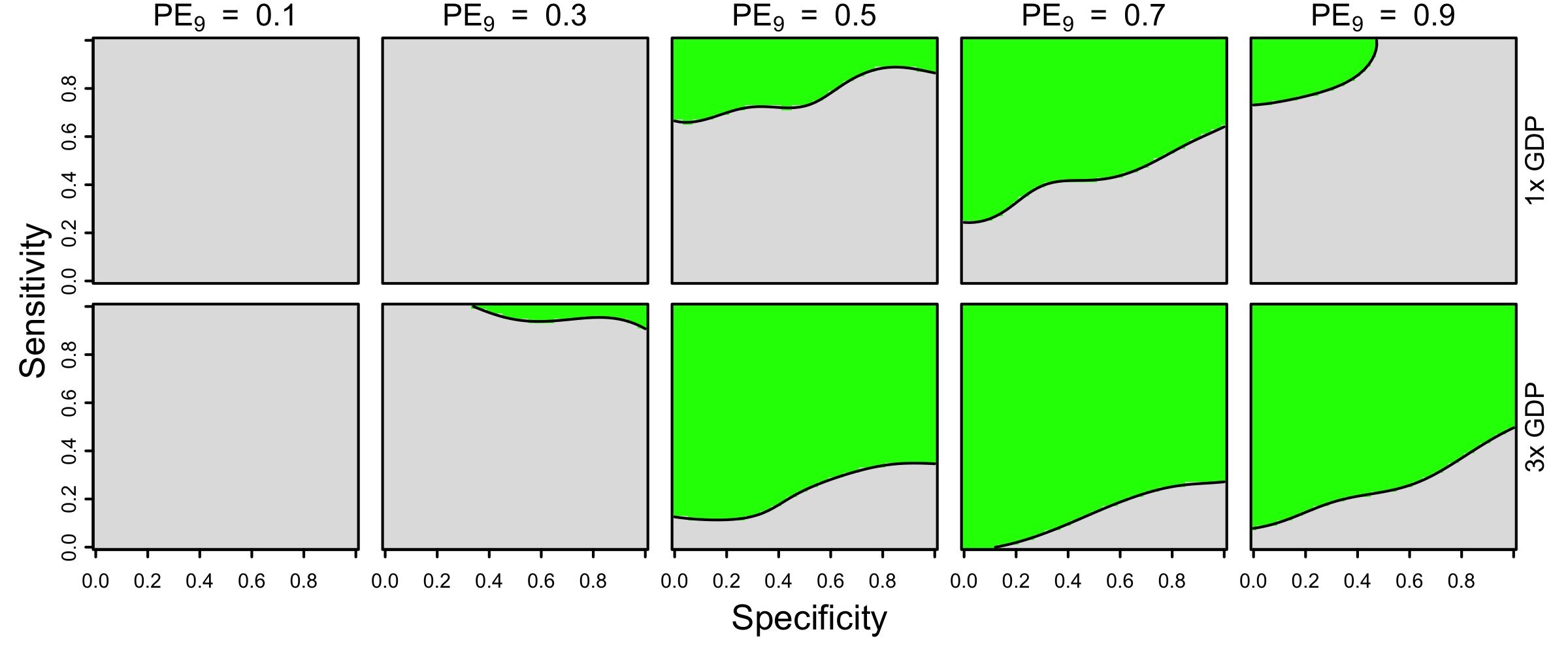

Supplement: S22 Fig — Cost-effectiveness according to Eq 5 is shown in green as a function of sensitivity (y-axis), specificity (x-axis), and PE9 value (columns). The value of costDALY is equal to per capita GDP (8,650 USD) in the top row and three times per capita GDP in the bottom row. (JPEG) [file pntd.0007482.s026.jpeg]

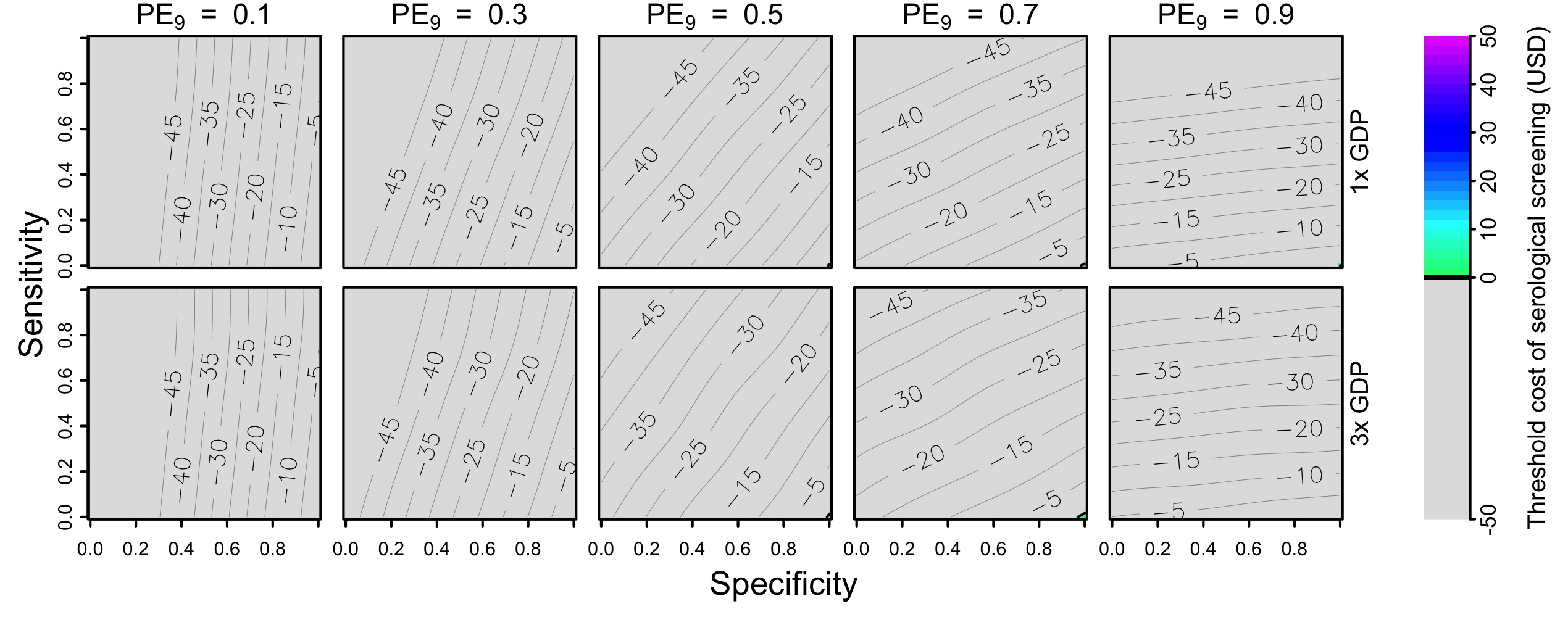

Supplement: S23 Fig — Threshold costs are indicated by color as a function of sensitivity (y-axis), specificity (x-axis), and PE9 value (columns). The value of costDALY is equal to per capita GDP (8,650 USD) in the top row and three times per capita GDP in the bottom row. (JPEG) [file pntd.0007482.s027.jpeg]
